# Physician Versus Large Language Model Chatbot Responses to Web-Based Questions From Autistic Patients in Chinese: Cross-Sectional Comparative Analysis

**DOI:** 10.2196/54706

**Published:** 2024-04-30

**Authors:** Wenjie He, Wenyan Zhang, Ya Jin, Qiang Zhou, Huadan Zhang, Qing Xia

**Affiliations:** 1 Tianjin University of Traditional Chinese Medicine Tianjin China; 2 Dongguan Rehabilitation Experimental School Dongguan China; 3 Lanzhou University Second Hospital Lanzhou University Lanzhou China; 4 Dongguan Songshan Lake Central Hospital Guangdong Medical University Dongguan China

**Keywords:** artificial intelligence, chatbot, ChatGPT, ERNIE Bot, autism

## Abstract

**Background:**

There is a dearth of feasibility assessments regarding using large language models (LLMs) for responding to inquiries from autistic patients within a Chinese-language context. Despite Chinese being one of the most widely spoken languages globally, the predominant research focus on applying these models in the medical field has been on English-speaking populations.

**Objective:**

This study aims to assess the effectiveness of LLM chatbots, specifically ChatGPT-4 (OpenAI) and ERNIE Bot (version 2.2.3; Baidu, Inc), one of the most advanced LLMs in China, in addressing inquiries from autistic individuals in a Chinese setting.

**Methods:**

For this study, we gathered data from DXY—a widely acknowledged, web-based, medical consultation platform in China with a user base of over 100 million individuals. A total of 100 patient consultation samples were rigorously selected from January 2018 to August 2023, amounting to 239 questions extracted from publicly available autism-related documents on the platform. To maintain objectivity, both the original questions and responses were anonymized and randomized. An evaluation team of 3 chief physicians assessed the responses across 4 dimensions: relevance, accuracy, usefulness, and empathy. The team completed 717 evaluations. The team initially identified the best response and then used a Likert scale with 5 response categories to gauge the responses, each representing a distinct level of quality. Finally, we compared the responses collected from different sources.

**Results:**

Among the 717 evaluations conducted, 46.86% (95% CI 43.21%-50.51%) of assessors displayed varying preferences for responses from physicians, with 34.87% (95% CI 31.38%-38.36%) of assessors favoring ChatGPT and 18.27% (95% CI 15.44%-21.10%) of assessors favoring ERNIE Bot. The average relevance scores for physicians, ChatGPT, and ERNIE Bot were 3.75 (95% CI 3.69-3.82), 3.69 (95% CI 3.63-3.74), and 3.41 (95% CI 3.35-3.46), respectively. Physicians (3.66, 95% CI 3.60-3.73) and ChatGPT (3.73, 95% CI 3.69-3.77) demonstrated higher accuracy ratings compared to ERNIE Bot (3.52, 95% CI 3.47-3.57). In terms of usefulness scores, physicians (3.54, 95% CI 3.47-3.62) received higher ratings than ChatGPT (3.40, 95% CI 3.34-3.47) and ERNIE Bot (3.05, 95% CI 2.99-3.12). Finally, concerning the empathy dimension, ChatGPT (3.64, 95% CI 3.57-3.71) outperformed physicians (3.13, 95% CI 3.04-3.21) and ERNIE Bot (3.11, 95% CI 3.04-3.18).

**Conclusions:**

In this cross-sectional study, physicians’ responses exhibited superiority in the present Chinese-language context. Nonetheless, LLMs can provide valuable medical guidance to autistic patients and may even surpass physicians in demonstrating empathy. However, it is crucial to acknowledge that further optimization and research are imperative prerequisites before the effective integration of LLMs in clinical settings across diverse linguistic environments can be realized.

**Trial Registration:**

Chinese Clinical Trial Registry ChiCTR2300074655; https://www.chictr.org.cn/bin/project/edit?pid=199432

## Introduction

Artificial intelligence (AI) has revolutionized human-computer interaction, reshaping communication, learning, and creativity paradigms [1,2]. A significant advancement in this realm is the emergence of large language models (LLMs), which have enabled the development of versatile digital assistants capable of understanding and generating human language [3-5]. Through extensive training on textual data, LLMs have acquired profound knowledge across diverse domains, facilitating coherent and contextually relevant interactions in natural language conversations. These models find applications in various domains, including natural language processing, question answering, language generation, and interactive dialogues [6-10]. Moreover, several studies have documented the use of LLMs in the medical field such as medication consultation [11], health education [12], and medical guidance [13,14].

Autism spectrum disorder (ASD) is a lifelong neurodevelopmental condition characterized by profound social and psychological challenges [15,16]. The estimated prevalence of ASD is approximately 1 in 36 children, with China reporting a prevalence of 1% [17-19], making it a significant public health concern. However, constraints on health care infrastructure development in China have led to resource shortages in numerous regions [20,21], exacerbating the burden on families and society. AI assistants represent underused resources for enhancing diagnosis and treatment efficiency in health care [22]. ChatGPT (OpenAI) [23] and ERNIE Bot (Baidu, Inc) [24] represent AI technologies powered by advancements in LLM. ChatGPT is a model with 20 billion parameters [25]. ERNIE Bot’s training data, as promoted at ERNIE Bot conference, includes trillions of web page data, billions of search data and image data, tens of billions of daily voice call data, and 550 billion facts of a knowledge graph, which distinguishes Baidu’s Chinese-language processing capabilities [26]. While ChatGPT gained widespread recognition for its ability to generate humanlike text across diverse topics [27,28], ERNIE Bot represents the forefront of AI technology in China [29]. Despite their original non–health care focus, their potential to assist in addressing patient inquiries remains unexplored mainly [30-32]. Implementing tiered diagnosis and treatment systems to optimize medical resource use may limit patients’ access to high-quality health care [33].

This study aims to investigate the performance of 2 conversational agents, ERNIE Bot and ChatGPT, in supporting individuals with ASD during web-based interactions. Our hypotheses are 2-fold. First, we hypothesize that ERNIE Bot, developed in China and trained on a data set that includes more Chinese text, may exhibit superior performance compared to ChatGPT, particularly regarding cultural relevance and linguistic nuances. Second, we anticipate that both ERNIE Bot and ChatGPT will demonstrate efficacy in assisting individuals with ASD, as evidenced by their ability to engage users effectively and provide helpful responses during conversational exchanges. Researchers have conducted numerous studies in English evaluating the benefits of LLMs in the medical field. Given the global significance of the Chinese language, this study aims to assess the capability of LLMs to provide high-quality and empathetic responses to health care queries posed by autistic individuals in China.

## Methods

### Data Source

This cross-sectional study aimed to construct a database of inquiries from autistic individuals by aggregating publicly available data from the web-based medical consultation platform DXY [34]. In China, chatbots are not permitted in clinical settings due to existing regulations, prompting the consideration of DXY as a feasible substitute. DXY is a prominent digital health care technology company with a 2-decade track record. The company offers a range of health-related applications, including high-quality health information dissemination, general knowledge services, a web-based medical consultation platform, health product e-commerce, and offline medical treatment. DXY caters to more than 100 million general users and has a user base of 5.5 million professionals, including 2.1 million physicians, constituting approximately 71% of the total number of physicians in the country.

The objective of this cross-sectional study was to analyze 200 cases to detect a 10% disparity (45% vs 55%) in responses provided by physicians and chatbots, with an assumed statistical power of 80%. We planned to use publicly accessible autism-related consultation records from the DXY website. Our sample comprised 100 randomly selected patients from the consultation records from January 2018 to August 2023. Each patient posed 1 to 3 questions, resulting in a total collection of 239 consultation queries. The qualifications of the responding health care professionals ranged from general to chief physicians.

### Ethical Considerations

We adhered strictly to the terms and conditions of DXY for all analyses, and the medical ethics committee of Lanzhou University Second Hospital approved them (approval 2023A-420). The study used publicly available data from the consultation platform, did not involve personal patient information or direct test subjects, and thus did not require informed consent. We registered the study on the Chinese Clinical Trial Registry (ChiCTR2300074655).

### Text Generation With an LLM Chatbot

To closely simulate an authentic medical consultation process, original questions were introduced into a new chatbot conversation from August 16, 2023, to August 30, 2023. In this dialogue, any questions previously posed with a potential impact on the outcomes were deliberately excluded. Both GPT-4 and ERNIE Bot 2.2.3 versions were used for this purpose. After eliminating expressions indicative of AI features, all responses were systematically collected and organized into a structured question-and-answer data set. The consultation content, which includes regional dialects and typographical errors, was carefully maintained to replicate a medical consultation authentically. The directly quoted content was used to prompt responses from the chatbot. The chatbot simulated a physician’s responses to mimic them while closely hiding its AI identity.

### Expert Evaluation

A team of 3 chief physicians specializing in child psychiatry and pediatric health care from distinct hospitals comprehensively reviewed the original questions and physicians’ and chatbot’s responses. The evaluators were presented with complete patient questions and physician and chatbot responses. The responders’ identities were anonymized; randomized; and labeled as responses 1, 2, or 3 to ensure that evaluators remained blinded. The evaluators were instructed to thoroughly examine the entire patient question and 3 responses before assessing the quality of the interactions. The evaluation process commenced with identifying the superior response and evaluating the responses across the 4 dimensions using a Likert scale: relevance, correctness, usefulness, and humaneness. The Likert-scale options for each dimension were as follows: relevance (irrelevant, somewhat relevant, partially relevant, relevant, or very relevant), correctness (incorrect, primarily incorrect, partially correct, correct, or very correct), usefulness (useless, of limited use, somewhat useful, useful, or very useful), and humaneness (lacking, slightly humane, moderately humane, humane, or very humane). Researchers assigned ratings on a 1-5 scale, with 1 representing the lowest quality and 5 representing the highest quality. Finally, a comparative assessment of the 3 responses was performed, with the quality dimensions for response evaluation detailed in Textbox 1.

**Quality dimensions in the comparative evaluation of responses.**

**Relevance**

- This dimension evaluates the alignment of responses with test results, emphasizing the system’s ability to generate appropriate text addressing specific issues rather than diverging into unrelated scenarios.

**Correctness**

- The correctness dimension focuses exclusively on the accuracy of information within the response, irrespective of the patient’s question. It gauges the scientific and technical precision of explanations based on best medical evidence and practices.

**Usefulness**

- This dimension combines the relevance and correctness of the system and evaluates its ability to provide non-obvious insights to patients, non-professionals, and laypersons. It includes providing appropriate recommendations, supplying relevant and accurate information, enhancing patient understanding of test results, and advising actions that optimize health care service use for the patient’s benefit.

**Empathy**

- Empathy involves demonstrating abundant respect, effective communication, compassion, and seeking emotional connections with patients. It encompasses recognizing and empathizing with their experience, respecting their thoughts, addressing their concerns patiently, and sincerely promoting their physical and mental well-being. Additionally, empathy entails humanely fulfilling patients’ and their families’ physical, psychological, social, and spiritual needs.

### Data Analysis

We used the Kruskal-Wallis H test to assess and compare the quality of the responses provided by physicians, ChatGPT, and ERNIE Bot along 4 dimensions: relevance, correctness, usefulness, and empathy. We presented the distribution of responses from each source including preferences for physicians, ChatGPT, and ERNIE Bot. Furthermore, we examined the proportion of responses that exceeded or fell below critical thresholds such as relevance, correctness, and usefulness. We compared the distribution of these threshold proportions among responses from physicians, ChatGPT, and ERNIE Bot. All statistical analyses were performed using SPSS (version 27.0; IBM), with a significance level set at *P*<.05 (2-tailed).

## Results

### Preferred Responses

This study included 717 evaluations of 239 randomly selected consultation questions. Evaluators indicated their preferences for physicians, ChatGPT, or ERNIE Bot at proportions of 46.86% (336/717; 95% CI 43.21%-50.51%), 34.87% (250/717; 95% CI 31.38%-38.36%), and 18.27% (131/717; 95% CI 15.44%-21.10%), respectively.

### Relevance

**Figure 1:**
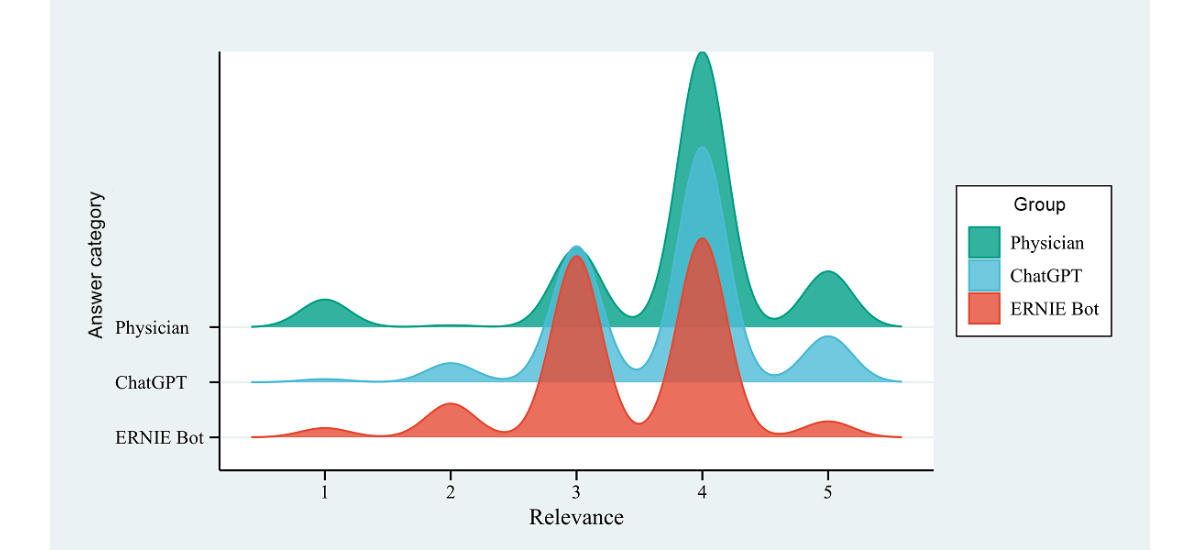
Distribution of relevance scores for responses to autistic patient questions by physicians and chatbots.

The distribution of the relevance scores among the 3 groups was not entirely uniform, exhibiting statistically significant differences (H=111.67, *P*<.001). Physician responses demonstrated higher relevance than chatbots (ChatGPT or ERNIE Bot). Specifically, the mean relevance score for physician responses was 3.75 (95% CI 3.69-3.82), whereas the mean relevance scores for ChatGPT and ERNIE Bot were 3.69 (95% CI 3.63-3.74) and 3.41 (95% CI 3.35-3.46), respectively (Figure 1). The proportion of responses rated as off topic (score <4) was lower for physicians (176/717, 24.55%; 95% CI 21.40%-27.70%) than for ChatGPT (258/717, 35.98%; 95% CI 32.47%-39.49%) and ERNIE Bot (366/717, 51.05%; 95% CI 47.39%-54.71%). Post hoc pairwise comparisons using the Bonferroni correction for significance levels revealed statistically significant differences in relevance scores between all 3 groups, specifically between physicians and ChatGPT (*P*<.001), physicians and ERNIE Bot (*P*<.001), and ChatGPT and ERNIE Bot (*P*<.001).

### Correctness

**Figure 2:**
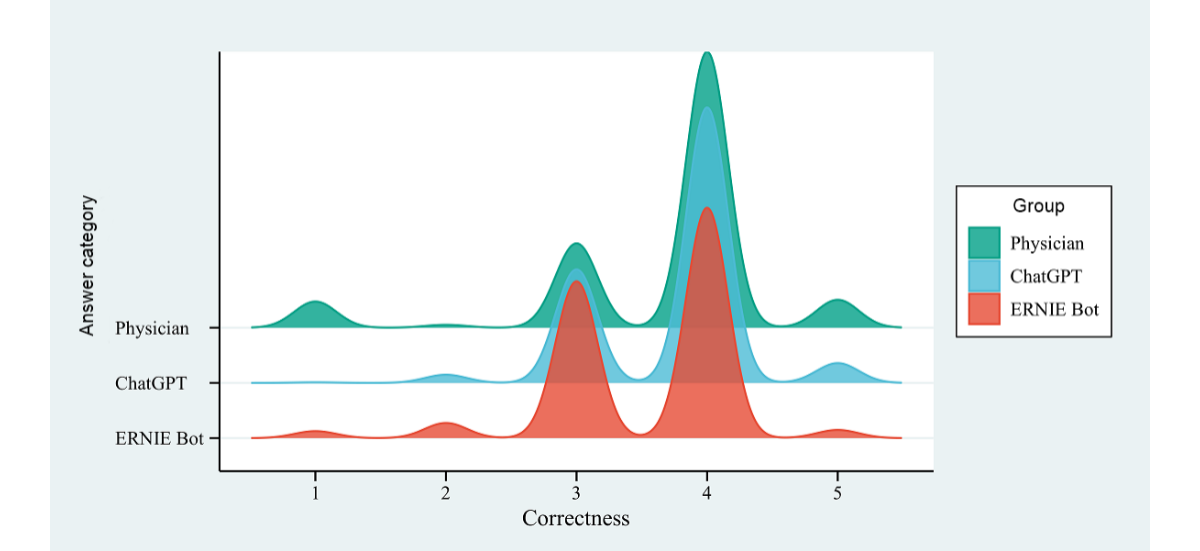
Distribution of correctness scores for responses to autistic patient questions by physicians and chatbots.

The mean correctness scores for physicians, ChatGPT, and ERNIE Bot were 3.66 (95% CI 3.60-3.73), 3.73 (95% CI 3.69-3.77), and 3.52 (95% CI 3.47-3.57), respectively (Figure 2). Physicians and ChatGPT achieved higher correctness scores among these 3 groups than ERNIE Bot. The distribution of the correctness scores exhibited statistically significant differences among the 3 groups (H=49.99, *P*<.001). When comparing the correctness scores between the 3 groups, the differences between physicians and ChatGPT (*P*=.58) were not statistically significant. However, the differences between physicians and ERNIE Bot (*P*<.001) and between ChatGPT and ERNIE Bot (*P*<.001) were both statistically significant. The proportion of responses with errors (score <4) was similar for physicians (196/717, 27.34%; 95% CI 24.08%-30.60%) and ChatGPT (211/717, 29.43%; 95% CI 26.09%-32.77%) and lower for ERNIE Bot (309/717, 43.10%; 95% CI 39.48%-46.72%).

### Usefulness

**Figure 3:**
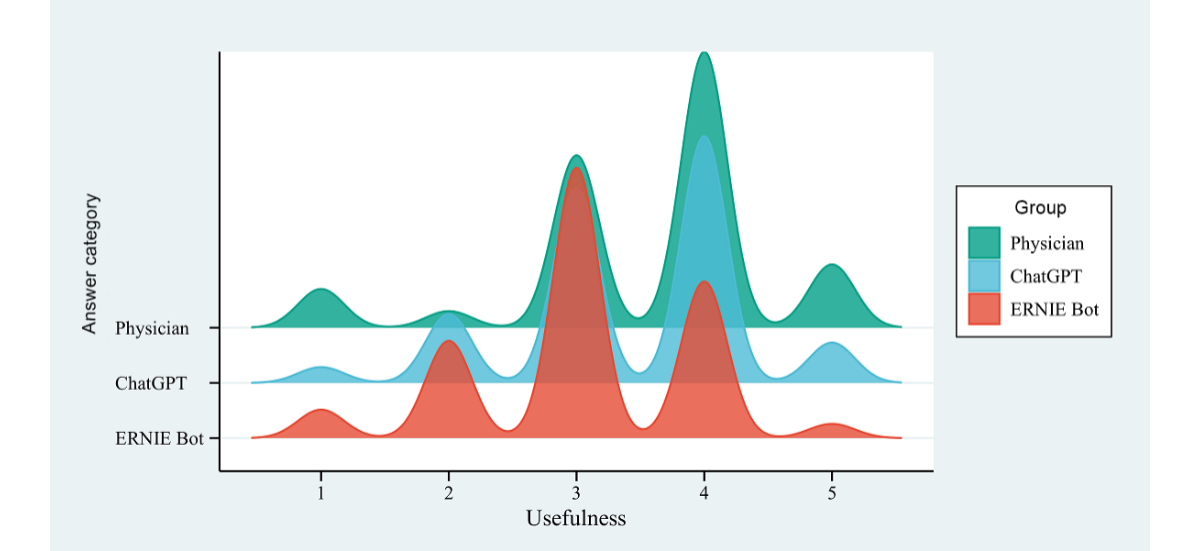
Distribution of usefulness scores for responses to autistic patient questions by physicians and chatbots.

Among the 3 response groups, physician responses exhibited higher levels of usefulness than chatbots (ChatGPT or ERNIE Bot). Specifically, the mean usefulness score for physician responses was 3.54 (95% CI 3.47-3.62), whereas the mean usefulness scores for ChatGPT and ERNIE Bot were 3.40 (95% CI 3.34-3.47) and 3.05 (95% CI 2.99-3.12), respectively (Figure 3). The proportion of responses rated as useful (score ≥4) was more significant for physicians (428/717, 59.69%; 95% CI 56.10%-63.28%) than for the chatbots (ChatGPT: 362/717, 50.49%; 95% CI 46.83%-54.15%; ERNIE Bot: 215/717, 29.99%; 95% CI 26.64%-33.34%). The distribution of the usefulness scores among the 3 groups displayed statistically significant differences (H=135.81, *P*<.001). Notably, all 3 pairwise comparisons of usefulness scores were statistically significant, with adjusted *P* values indicating significance, specifically between physicians and ChatGPT (*P*<.001), physicians and ERNIE Bot (*P*<.001), and ChatGPT and ERNIE Bot (*P*<.001).

### Empathy

**Figure 4:**
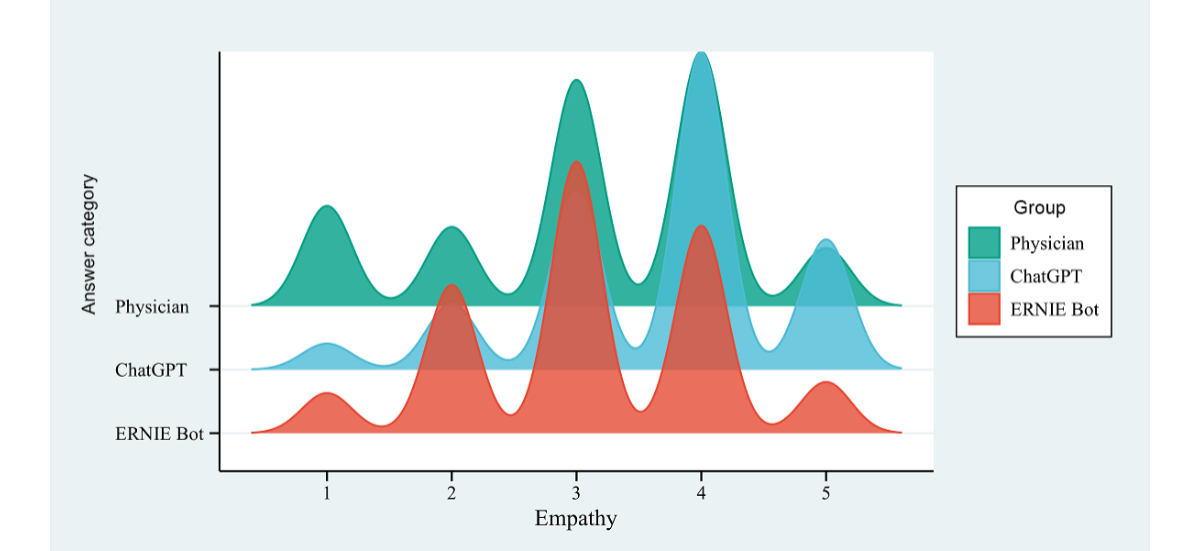
Distribution of empathy scores for responses to autistic patient questions by physicians and chatbots.

The mean empathy score for ChatGPT was 3.64 (95% CI 3.57-3.71), whereas the mean empathy scores for physicians and ERNIE Bot were 3.13 (95% CI 3.04-3.21) and 3.11 (95% CI 3.04-3.18), respectively (Figure 4). ChatGPT’s responses received higher empathy scores than physicians and ERNIE Bot within the 3 response groups. Specifically, the proportion of responses displaying empathy (score ≥4) was higher for ChatGPT (447/717, 62.34%; 95% CI 58.79%-65.89%) than for physicians (312/717, 43.51%; 95% CI 39.88%-47.14%) and ERNIE Bot (258/717, 35.98%; 95% CI 32.47%-39.49%). The distribution of empathy scores among the 3 groups revealed significant differences (H=118.58, *P*<.001). When assessing empathy scores among the 3 groups, the differences between physicians and ChatGPT (*P*<.001) and between ChatGPT and ERNIE Bot (*P*<.001) both demonstrated statistical significance. However, the disparities between physicians and ERNIE Bot (*P*=.14) were not significant.

The evaluators performed a reliability assessment, which revealed robust repeatability. The intraclass correlation coefficient values for the 4 response categories (relevance, correctness, usefulness, and empathy) were 0.812 (*P*<.001), 0.831 (*P*<.001), 0.818 (*P*<.001), and 0.863 (*P*<.001), respectively.

## Discussion

### Principal Findings

This study evaluated the capabilities of LLMs, such as ChatGPT and ERNIE Bot, in delivering quality and empathetic responses to medical queries from Chinese autistic individuals. To simulate genuine clinical scenarios, publicly accessible data from web-based medical consultation platforms were used in this cross-sectional investigation. Notably, prevailing regulations in China strictly prohibit the use of AI-generated prescriptions. The study results revealed that expert evaluators favored responses from physicians over those generated by chatbots (ChatGPT or ERNIE Bot). In contrast to previous ophthalmology research that demonstrated chatbots outperforming physicians, our findings indicated that physicians received higher scores in relevance, correctness, and usefulness, with only a slight margin behind ChatGPT in terms of empathy. Conversely, ERNIE Bot obtained the lowest scores across all 4 dimensions: relevance, correctness, usefulness, and empathy.

Bernstein et al [35] compared the occurrence of incorrect or inappropriate content, potential harm, and degree of harm in responses from chatbots and humans. The study indicated that chatbot responses exhibited a similar likelihood of containing incorrect or inappropriate material compared with human responses (prevalence ratio [PR] 0.92, 95% CI 0.77-1.10). Moreover, no significant differences were observed between chatbot and human responses regarding potential harm (PR 0.84, 95% CI 0.67-1.07) or the degree of harm (PR 0.99, 95% CI 0.80-1.22). These results suggested that LLMs can provide appropriate ophthalmological advice for varying levels of complexity in patient questions. Shao et al [36] developed a set of 37 questions for patient education on thoracic surgery during the perioperative period, covering topics such as disease information, diagnostic procedures, perioperative complications, treatment measures, disease prevention, and perioperative care instructions. An assessment of responses in both English and Chinese contexts revealed that 92% (n=34) were considered appropriate and comprehensive. This study highlighted the potential feasibility of using ChatGPT for patient education in thoracic surgery in both English and Chinese settings. Zhu et al [37] investigated the application of ChatGPT as a mediator between physicians and patients in Chinese-speaking outpatient settings, focusing mainly on the Chinese Physician Qualification Examination. The study reported an average score of 72.4% in the clinical knowledge section, which was placed within the top 20 percentile. These findings suggested that ChatGPT can facilitate physician-patient communication in Chinese-speaking outpatient settings. Ayers et al [38] used publicly available data from a social media forum, Reddit, to compare physician responses and the ChatGPT. They randomly selected 195 dialogues with questions answered by physicians and generated chatbot responses by inputting the original questions into a new ChatGPT session. Evaluators assessed the responses from both physicians and the ChatGPT using a Likert scale, considering preferences, information quality, and empathy or interpersonal style. In 585 evaluations, 78.6% (95% CI 75%-81.8%) of evaluators preferred chatbot responses over those from physicians. These studies highlight the significant potential of chatbots in the field of medicine.

In our study, which consisted of 717 evaluations, evaluators preferred physician responses over those from chatbots, namely ChatGPT or ERNIE Bot. The preferences for physicians, ChatGPT, and ERNIE Bot were 46.86% (n=336; 95% CI 43.21%-50.51%), 34.87% (n=250; 95% CI 31.38%-38.36%), and 18.27% (n=131; 95% CI 15.44%-21.10%), respectively. Physician responses achieved higher scores in relevance, accuracy, and usefulness, with the only exception being the dimension of empathy, which ChatGPT surpassed. Our cross-sectional study’s results differed from previous reports due to several factors. First, unlike previous research primarily conducted in English-speaking settings, this study was conducted in a Chinese-speaking environment. Second, we preserved the original medical questions from autistic patients without modification to simulate authentic clinical consultations, including errors or nonstandard expressions in the queries such as misspellings or dialects. Our study contrasted with previous research, which often relies on professionally standardized queries. Furthermore, autistic patients’ queries frequently entail subjective matters such as seeking recommendations for specialist physicians or autism-related resources, lacking standardized responses, and reflecting significant cultural variations. Finally, our study used samples from a paid web-based medical consultation platform, whereas Ayers et al [38] used dialogues from public social forums on Reddit. Physicians may exhibit more proactive and diligent engagement in paid consultations.

Our study compared the performance of ChatGPT and ERNIE Bot in physician-patient interactions, with ERNIE Bot trained in Chinese and ChatGPT in English. While one might assume that ERNIE Bot’s training in Chinese would result in greater empathy toward Chinese-speaking users than ChatGPT, the results challenge this notion. The findings suggest that factors beyond the language of training influence the empathetic responsiveness of LLMs, highlighting the complexity of human-AI interactions and emphasizing the need for further exploration of the relationship between language and empathy. Physicians responded better when patients asked for recommendations on specific Chinese books about ASD. Physicians also effectively handled situations where the patient’s condition was misstated, whereas LLMs provided inaccurate information. Additionally, creating user-friendly interfaces to accommodate patients with varying levels of technological proficiency could improve the accessibility and usability of AI models in health care settings.

### Limitations

This study had several notable limitations. First, reliance on web-based consultation platform records constrained each autistic patient to a maximum of 3 questions per web-based consultation, potentially limiting the ability to replicate real-world patient-physician interactions comprehensively. Moreover, the study exclusively examined text-based responses to patient inquiries, neglecting the potential for health care professionals to tailor their responses based on individual patient characteristics such as occupation and emotional state. The extent to which clinical professionals can adapt their responses to such personalization remains uncertain. Additionally, the study did not assess the chatbot’s capacity to extract information from health records, representing an area of potential improvement. Finally, although the evaluators have been single blinded, they potentially introduced bias into their assessments because they are coauthors of the paper and may hope that there are apparent differences between the groups in the results to make extreme scores.

### Conclusions

The findings of this cross-sectional study show that physician responses outperform those of LLMs in the Chinese context, responding to inquiries from autistic patients in text-based formats compared with responses from current state-of-the-art LLMs. Nevertheless, these LLMs can offer medical guidance for autistic patients and demonstrate greater empathy compared with physicians, particularly ChatGPT-4. It is essential to emphasize that further refinement and comprehensive research are prerequisites before deploying LLMs effectively in clinical settings across diverse linguistic environments.

## Appendix

Multimedia Appendix 1 The raw data.

## Abbreviations

AI: artificial intelligence
ASD: autism spectrum disorder
LLM: large language model
PR: prevalence ratio

## Data Availability

All data generated or analyzed during this study are included in this published article. The data for this study are contained in Multimedia Appendix 1.
